# The Implementation of Mass-Vaccination against SARS-CoV-2: A Systematic Review of Existing Strategies and Guidelines

**DOI:** 10.3390/vaccines9040326

**Published:** 2021-04-01

**Authors:** Tasnim Hasan, Justin Beardsley, Ben J. Marais, Thu Anh Nguyen, Greg J. Fox

**Affiliations:** 1 Faculty of Medicine and Health, The University of Sydney, Sydney, NSW 2006, Australia; tasnim.hasan@sydney.edu.au (T.H.); justin.beardsley@sydney.edu.au (J.B.); 2 The Woolcock Institute of Medical Research, Glebe, NSW 2037, Australia; thuanh.nguyen@sydney.edu.au; 3 Marie Bahir Institute, The University of Sydney, Westmead, NSW 2145, Australia; ben.marais@health.nsw.gov.au

**Keywords:** covid, vaccination, national policy data, implementation

## Abstract

The global drive to vaccinate against severe acute respiratory syndrome-coronavirus-2 (SARS-CoV-2) began in December 2020 with countries in Europe, Middle East, and North America leading the roll out of a mass-vaccination program. This systematic review synthesised all available English-language guidelines and research regarding mass-vaccination for COVID-19 until 1 March 2021—the first three months of the global mass-vaccination effort. Data were extracted from national websites, PubMed, Embase, Medline and medRxiv, including peer and non-peer review research findings. A total of 15 national policy documents were included. Policies were summarised according to the World Health Organisation (WHO) framework for mass vaccination. All included policies prioritised front-line health care workers and the elderly. Limited information was available regarding staffing, cold chain, communication strategies and infrastructure requirements for effective vaccine delivery. A total of 26 research studies were identified, reporting roll-out strategies, vaccine uptake and reasons for refusal, adverse effects, and real-life estimates of efficacy. Early data showed a reduction in SARS-CoV-2 cases, hospitalisation and deaths in settings with good coverage. Very low rates of vaccine-related serious adverse events were observed. These findings provide an overview of current practice and early outcomes of COVID-19 mass-vaccination, guiding countries where roll-out is yet to commence.

## 1. Introduction

By the end of January 2021, the coronavirus disease-2019 (COVID-19) pandemic caused by severe acute respiratory syndrome-coronavirus-2 (SARS-CoV-2) was responsible for more than 100 million infections and 2.5 million deaths globally [1]. Vulnerable communities and ethnic minorities have shouldered particularly high physical, psychological, social, and economic burdens [2,3]. While the implementation of border restrictions, social distancing and infection control practices has curtailed the pandemic in some settings, such measures do not provide a feasible long-term solution, given that SARS CoV-2 has become an endemic virus [4].

Vaccination has the potential to substantially reduce the incidence of severe disease, morbidity, and mortality, especially if "herd immunity" can be attained. By January 2021, more than 10 vaccines were in production, utilising a range of established and new vaccine technologies, including novel mRNA approaches [5]. The variable efficacy reported with different vaccines has been the source of much scientific debate and media speculation. However, with regards to vaccine acceptance and uptake amongst the public, government trust and implementation strategies have been shown to be more important than objective measures of vaccine efficacy [6,7]. Despite the excellent progress made in some countries, equity of access to vaccines by vulnerable populations remains a challenge—particularly those in low- and middle-income countries. Without an effective global vaccination response, vulnerable populations will continue to experience preventable morbidity, economic recoveries are likely to stall, and border closures will remain.

The drive to vaccinate large populations began in earnest in mid-December 2020 in Europe, the Middle East, and North America [8]. By the end of February 2021, Israel had vaccinated over 80% of its population, presenting a model for rapid implementation [8]. Israel’s success has been attributed to high-level political commitment together with coordinated and well-resourced collaboration between the government and health care providers [9]. Despite this early progress, more than 100 countries are yet to commence vaccination [8]. The WHO has produced a framework for mass vaccination policies, including the following domains: coordination, planning, vaccination strategies, access, and community engagement [10]. However, few national plans for vaccine roll-out have been made publicly available—most of which were from high-income settings [11,12]. Dissemination of existing national guidelines for mass-vaccination will assist countries to develop their own local strategies.

This rapid review assessed the publicly-available policies and implementation strategies used for COVID-19 mass vaccination. It characterises differences in national vaccine policies and evaluates determinants of successful scale-up, so that countries currently developing mass-vaccination strategies can benefit from the experiences in countries where mass-vaccination has already begun. 

## 2. Materials and Methods

**Figure 1:**
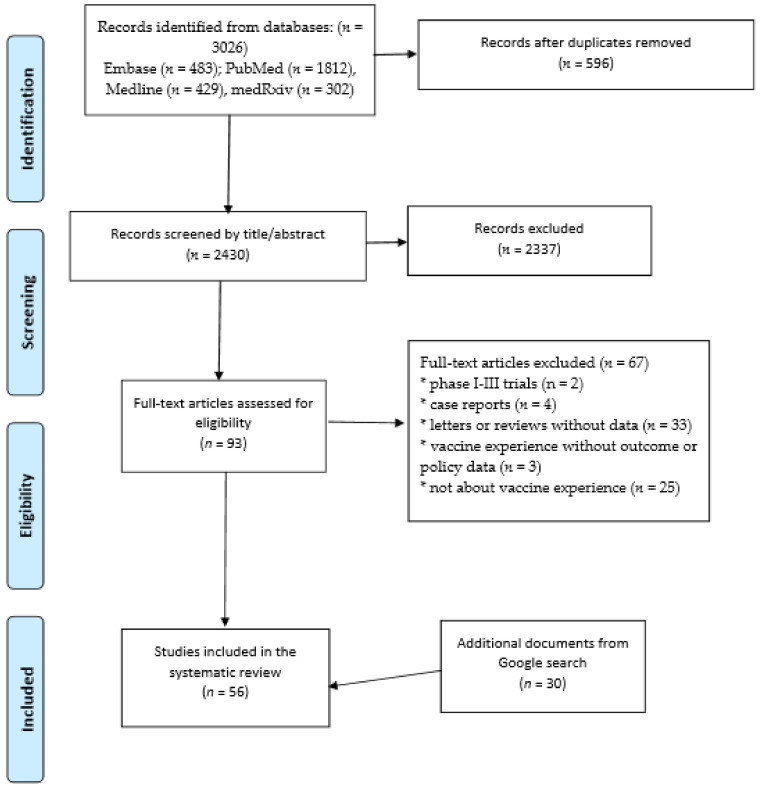
PRISMA Flow diagram for study inclusion in the systematic review

We reviewed all literature available until the 1st of March 2021. As this is a rapidly evolving area, articles from both peer-reviewed and selected non-peer reviewed online sources were included. The review was completed in accordance with the Preferred Reporting Items for Systematic review and Meta-Analysis (PRISMA) guidelines (Figure 1) [13]. 

### 2.1. Search Strategy—Research Articles

We performed a systematic search of the PubMed, Medline, Embase and medRxiv databases using the following terms for title/abstract: “SARS-CoV-2”, “covid*”, “coronavirus” and “vaccine”. The search strategy was restricted to articles published between the 10th of December (the approximate timing of the commencement of vaccination) and the 1st of March. Articles were imported into Endnote X9.3.2 (Clarivate Analytics), and duplicates were removed. All types of articles from all countries were considered acceptable for inclusion, provided they described real-life experience with vaccine rollout. Non-English language articles, case-reports, Phase 2 or Phase 3 clinical trials, and vaccine efficacy trials were excluded as they did not reflect large-scale vaccine deployment. Title and abstract screening for articles was performed to exclude articles not meeting the inclusion criteria. The remaining articles underwent full-text review for final inclusion. Articles in the reference lists of included papers were also screened for inclusion.

### 2.2. Search Strategy: Non-Peer Reviewed Literature

A search of "grey" (unpublished) literature was completed on 1 March 2021. National and health websites for United Kingdom (UK), European Union (EU), United States of America (USA), Israel, Canada, India, China, and Russia were searched. Additionally, data were sought from high-income, middle-income, and low-income countries [14], from each World Health Organization (WHO) region. Only English language sources were included. Documents that were not official government or related to national vaccine campaigns were excluded (e.g., sub-national policies). The most recent version of documents was included.

The Google.com search was used to identify publicly available national policy documents and reports of vaccine outcomes for all 85 countries that had commenced vaccinations by the March 1st, 2021 [8]. The following terms were used:

“[Country name]” COVID-19 vaccination policy

“[Country name]” COVID-19 vaccination delivery plan

“[Country name]” COVID-19 vaccination progress

“[Country name]” COVID-19 vaccination tracker

Country profiles in “Our World in Data” [8], a website which provides a live tracker of global vaccination status, were interrogated for articles which met inclusion criteria. 

Data sources requiring clarification were reviewed by three different authors (TH, JB, GF) with a majority decision made about inclusion.

### 2.3. Data extraction and Analysis

Extracted data included population demographics, vaccination strategy, implementation challenges and vaccination outcomes. National policy documents were reviewed according to the WHO framework for vaccination [10]. Data was entered into Microsoft Excel. Findings were also synthesised narratively.

### 2.4. Ethical Issues

Ethical approval was not required for this search of publicly available documents.

## 3. Results

Our combined searches retrieved 3026 articles, of which 596 were duplicates and 2337 were excluded after title and abstract review (Figure 1). A total of 93 full-text articles were reviewed and 26 were included. Thirteen articles were from peer-reviewed sources and 13 were non-peer reviewed articles. An additional 30 online reports were also included. No additional data sources were identified by searching reference lists.

The review identified 15 national policy documents (Table 1) and 15 reports of vaccine outcomes summarising outcome data (Table S1). Findings from research article are summarised in Table 2. 

### 3.1. National Policy Documents

Out of 85 countries searched using Google.com, 71 did not have English language documents available. One document described policy for the European Union as a whole, with additional national policy documents located from different EU countries not in English. Of nine countries in Africa where vaccination had commenced, only two had English language documents (South Africa and Seychelles). Data from Seychelles was available through a government Facebook page. Two countries had twitter information in non-English language formats (Senegal and Zimbabwe). One other document was available in a non-English format (Morocco). Four English-language documents detailed policy information and vaccination priority groups via a question-and-answer format on national websites (Czech Republic, Qatar, Singapore, Monaco) [15,16,17,18].

Fifteen national policy documents were included. Fourteen came from high-income settings, one came from an upper-middle income setting (Lebanon), and none were identified from lower-middle, or lower income settings [11,12,15,16,17,18,19,20,21,22,23,24,25,26,27] (Table 1). One document was published from the European Union (EU) and encompassed a description of policies for 26 EU member countries [21]. Vaccination was not mandated in any setting. All documents stipulated that COVID-19 vaccination programs were publicly-funded. In Switzerland, mandatory individual private health insurance provided funding in the first instance, before public funding was used [20]. In Lebanon, it was anticipated that COVAX, vaccines would be supplied by the GAVI, the Vaccine Alliance (GAVI), and WHO [24].

Only three national policy documents reported policies in all domains included in the WHO framework for mass-vaccination [12,21,24]. Communication strategies were only listed in six documents [10,11,12,21,22,24], strategies included a "rumour tracking system" in Lebanon [24] and an online fact checker in some European settings [21].

### 3.2. Vaccine Deployment

The most common vaccines deployed were the vaccines developed by Pfizer, Moderna and AstraZeneca. Only five national policy documents [12,21,24,25,26] specified how they would manage the cold-chain requirements for the vaccines—in particular, Pfizer BioNTech and Moderna, which must be maintained at -80 degrees Celsius [12]. The policy document from Lebanon, stipulated a central storage site for the vaccine with cold-chain courier capacity [24]. New Zealand have procured nine central freezers with courier capacity [26] and the USA have multiple fridges in various locations [12].

Most national policy documents have not yet detailed problems with the vaccination drive. The EU policy document reported problems with extracting the final dose out of six-dose Pfizer vaccine vials, including a shortage of "dead space" syringes [21]. Belgium reported the total number of “wasted doses” on their website and at the end of February this was approximately equivalent to 16,000 doses (2% of total doses) [28].

The UK and two countries in the EU specified an acceptance of increased dosing interval for multi-dose vaccines [21,22]. In the UK despite having almost 30% vaccinated with the first dose of a COVID-19 vaccine, only 1.1% have received their second dose [29].

### 3.3. Vaccination of Priority Groups

All but three national policy documents provided a prioritisation strategy for the vaccination of vulnerable groups [10,11,12,15,18,19,20,21,22,23,24,25,27]. Six countries simultaneously prioritised both health care workers (HCW) at risk of exposure to the virus, as well as elderly individuals in care facilities [11,15,18,19,21,24]. Three countries prioritised frontline HCWs over elderly individuals in facilities [10,12,23] and three prioritised the elderly in facilities over frontline HCWs [20,22,27]. Three national policy documents discussed the prioritisation of those in institutional care, or younger people with chronic conditions [19,20,21]. Canada and Australia have indicated a priority strategy for Indigenous communities [11,19]. No national policy document recommended the vaccination of children under 16 years of age.

### 3.4. Infrastructure and Staffing for Vaccination

Vaccination was described to be mostly taking place in large vaccination hubs, such as sporting venues and other entertainment venues. Only six guidelines discuss the additional staffing, training, and recruitment requirements to achieve mass vaccination delivery [12,21,22,24,25,26].

### 3.5. Peer-Reviewed Research of Vaccination Outcomes

Of the 26 included peer-reviewed articles, ten were from Israel [30,31,32,33,34,35,36,37,38,39] and eight from the USA [40,41,42,43,44,45,46,47,48]. Six articles looked at adverse event data [42,43,44,45,49] and five looked at vaccine acceptance [46,47,50,51,52].

Several studies, mostly set in Israel look at vaccination outcomes on COVID-19 diagnosis, hospitalisation and mortality. All these studies confirm a >50% reduction in all of these values, with higher rates of reduction for those studies which look at outcomes after second dose [35]. Two Israeli studies report cases of incident COVID-19 after vaccination [31,37]. One of these demonstrated an average SARS-CoV-2 viral load in these individuals to be lower compared to the unvaccinated populations [37]. Two studies suggest that the immunological response is greater after vaccination, in those who have previously had COVID-19 [30,48].

**Table 1:** Summary of publicly-available national policy documents for the implementation of mass-vaccination against COVID-19.

Setting/Country	Australia	USA	Czech Republic	Qatar	Singapore	Monaco	Canada	Switzerland	EU	UK	South Africa	Lebanon	Cyprus	New Zealand	Ireland
Date of most recent update	17 February 2021	29 October 2020	10 February 2021	NR	26 February 2021	23 February 2021	25 February 2021	26 February 2021	1 February 2021	11 January 2021	NR	28 January 2021	2020	22 December 202	24 February 2021
Reference	[11]	[12]	[15]	[16]	[17]	[18]	[19]	[20]	[21]	[22]	[23]	[24]	[25]	[26]	[27]
Funding source	Public	Public	Public	Public	Public	Public	Public	Public	Public	Public	Public	Public	Public	Public	Public
				Pf/B Mod		Pf/B		Private			AZ/Ox	COVAX		NR	
Vaccine used	Pf/B	NR	NR	NR	Pf/B	No	Pf/B	Pf/B	Pf/B	Pf/B	NR	NR	NR	NR	Pf/B
				NR	Mod	NR		Mod	Mod		NR			NR	Mod
	AZ/Ox								AZ/Ox	AZ/Ox					AZ/Ox
Vaccination mandatory for target populations?	No	NR	NR	NR	No		No	No	No	No		No	NR	NR	No
Was an increased delay between the two doses recommended (compared to the frequency in published trials)	NR	NR	NR		NR	Priority 1	NR	NR	In 2 countries	Yes	Priority 1	NR	NR		NR
EQUITABLE ACCESS						Priority 1					Priority 2				
Priority criteria for vaccination					NR	Priority 1									
Frontline HCW	Priority 1	Priority 1	Priority 1			Priority 1	Priority 1	Priority 2	Priority 1	Priority 2	Priority 2	Priority 1	Priority 1		Priority 2
Chronic comorbidities	Priority 2	Priority 2	Priority 2				Priority 1	Priority 1	Priority 1	Priority 4			Priority 4		
Elderly in aged care	Priority 1	Priority 2	Priority 1				Priority 1	Priority 1	Priority 1	Priority 1		Priority 1			Priority 1
Other elderly	Priority 2	Priority 2	Priority 1				Priority 1	Priority 1	Priority 1	Priority 2	Priority 2	Priority 1	Priority 3		
Institutionalised		Priority 3					Priority 2	Priority 4			Priority 2		Priority 5		
Indigenous population	Priority 2	Priority 3					Priority 1								
Other HCW		Priority 2	Priority 2	NR		NR	Priority 1				NR		Priority 2	NR	
Essential public services	Priority 3		Priority 2	NR		NR					NR			Yes	
Children															
Expected date when whole adult population vaccinated	NR	NR	NR		NR		Sep 2021	NR	NR	NR	NR	NR	Mid 2022	NR	NR
Will extra doses be donated to COVAX	Yes	NR	NR		NR	Yes	NR	Yes	NR	Yes		NR	NR		NR
VACCINATION STRATEGIES															
Location of vaccination			NR		NR		NR	NR							
Large public venues	Yes	Yes		Drive through		Care home			Yes	Yes		Yes	Yes		Yes
Hospitals		Yes		NR		NR				Yes	NR			Electronic	
Clinics and pharmacies Other	Yes	Yes		NR		Self-reported to clinic, hotline, or email			Yes	Yes	NR		Yes	NR	Yes
Vaccination record (electronic/paper)	NR	NR	NR		NR		NR	Paper	Electronic	Electronic		Electronic	Electronic		NR
Adverse-effect reporting (automated electronic, electronic self-report, GP self-report)	Active surveillance via electronic prompts	NR	NR	NR	Self-reported to local doctor	NR	NR	NR	5 countries with self-reported electronic system	Review of electronic health records	NR	Self-reported electronic system	NR	9 extra freezer procured	NR
COORDINATION															
Cold chain infrastructure	NR	Extra freezer procured	NR	NR	NR	NR	NR	NR	Extra freezer procured	NR	NR	1 freezer procured	Freezer available		NR
PLANNING														Yes	
Increased labour requirements			NR		NR		NR	NR						Yes	NR
Non-medical staff employed for vaccination	Yes	Yes							Yes	Yes		Yes	Yes		
Training provided to staff	Yes	Yes		NR		NR			Yes	Yes	NR	Yes		NR	
COMMUNITY ENGAGEMENT															
Are the following employed?			NR		NR		NR	NR					NR		NR
Community strategies		Yes							Yes			Yes			
Social media		Yes							Yes			Yes			
Media campaigns	Yes	Yes		NR		NR			Yes	Yes	NR	Yes		NR	
Other				**Qatar**		**Monaco**			Celebrities		**South Africa**			**New Zealand**	
Strategies to reduce misinformation	NR	NR	NR	NR	NR	23 February 2021	NR	NR	Monitor media, online fact checker	NR	NR	Rumour tracking team	NR	22 December 202	NR

AZ/Ox AstraZenca/Oxford, EU European Union, GP general practitioner, HCW health care worker, Mod Moderna, NR not reported, Pf/B Pfizer BioNTech, UK United Kingdom, USA United States of America.

**Table 2:** Published peer-reviewed and non-peer reviewed articles summarising real-life experience with vaccination against SARS-CoV-2.

Country/Reference	Peer Reviewed (Yes/No)	Number Vaccinated in Study	Age Groups Immunised	Female (%)	Vaccines Used (% *)	Outcomes
Studies reporting demographic details						
USA **Gharpure et al. [40]**	Yes	713,909 (LTCF residents) 582,104 (HCW)	NR	NR	NR	Estimated 77.8% residents and 37.5% staff in 11,460 care facilities, vaccinated by Jan 17 (at least one dose)
USA **Painter et al. [41]**	Yes	12,928,749	<0.1% <18 70.9% 18–64 29% ≥65	63	Pf/B Mod	
Studies reporting adverse events						
Brazil **Pagotta et al. [53]**	No	683 (HCW)	99.3% 18–60 0.7% >60	68	Gamaleya	Adverse effects reported by 71.3% in a survey. Most common—injection site pain, myalgia, fever
India **Jayadevan et al. [49]**	No	5396	NR	NR	BB (95) AZ/Ox (3.3) Pf/B (0.8) Sinopharm (0.8)	Adverse effects reported by 65.9% in a survey, highest in those 20–39 years of age and females. Most common adverse effects—lethargy, myalgia, fever
USA **CDC [42]**	Yes	4,041,396	NR	NR	Mod	Anaphylaxis reported in 0.0002% (*n* = 10), serious adverse effects in 0.03% (*n* = 1266)
USA **CDC [43]**	Yes	1,893,360	NR	NR	Pf/B	Anaphylaxis reported in 0.001% (*n* = 21), serious adverse effects in 0.2% (*n* = 4393)
USA **Gee et al. [44]**	Yes	13,794,904	0.2% 0–17 90.3% 18–64 6.2% ≥65	61	Pf/B Mod	Adverse effects reported in 0.05% (*n* = 6994) through a passive national surveillance system
USA **McMurray et al. [45]**	No	31,029	NR	NR	Pf/B Mod	Review of electronic medical records found a 2.1 to 1500 times reduced frequency of adverse events reported when events were obtained from self-reported health interactions compared to active solicitation in trials or in post-marketing surveillance
Studies reporting vaccine acceptance						
Saudi Arabia **Barry et al. [50]**	No	352 (HCW)	91.9% 20–50 8.1% >50	57	Pf/B	Factors associated with not enrolling for vaccine: female, younger age, use of social media, foreign national. Percentage not yet registered for vaccine: 66.7% (*n* = 706)
UK **Martin et al. [51]**	No	12,278 (HCW)	18.7% <30 72% 30–60 9.3% >60	76	Pf/B AZ/Ox	Factors associated with lower vaccine uptake: ethnic minority, younger age, female, lower socio-economic status
UK **Kim [52]**	No	66,994	NR	NR	NR	Factors associated with vaccine uptake—pre-pandemic income, education. Least likely to take up vaccination—Black Hispanics
USA **Pamplona, Sullivan, Kotanko [46]**	Yes	115 (HCW)	NR	NR	NR	Factors associated with not being vaccinated in a dialysis ward (26.8%, *n* = 42)—past COVID-19, pregnancy, absence. 3.8% (*n* = 6) declined vaccine
USA **Schradering et al. [47]**	Yes	1136 (HCW)	98.6% 22–64 1.4% ≥65	59	NR	14% (*n* = 195) HCWs refused vaccination—usually for concern about adverse effects. Ethnic group most likely to decline vaccine: with non-Hispanic black HCWs
Studies reporting efficacy (excluding Phase 2 and 3 trials of vaccine efficacy)						
Israel **Abu Jabal et al. [30]**	Yes	514	2.1% <30 79.4% 30–59 18.5% ≥60	63	Pf/B	Immunogenicity post vaccination similar by ethnicity and sex, but decrease with age. Increased immunogenicity in previous COVID-19 cases
Israel **Amit et al. [31]**	Yes	4081	NR	NR	Pf/B	In vaccinated HCW, 0.54% (*n* = 22) developed COVID-19 within 10 days of vaccination
Israel **Amit et al. [32]**	Yes	NR	NR	NR	NR	30% and 75% reduction in SARS-CoV-2 cases in vaccinated HCW vs. unvaccinated HCW 14 and 28 days, respectively after vaccination
Israel **Aran [33]**	No	NR	NR	NR	Pf/B	72% reduction in cases and 83% reduction in hospitalisation by modelling
Israel **Chodick et al. [34]**	No	503,875	Mean 59.7	52	Pf/B	Cases of COVID-19 infection 24 days post vaccination: 0.84% (*n* = 3098) 51% vaccine effectiveness calculated after 1st dose
Israel **Dagan et al. [35]**	Yes	596,618	72% <60 28% ≥60	50	Pf/B	92% reduction in COVID-19 cases, 87% reduction in hospitalisation, 72% reduction in deaths: at 7 days after second dose
Israel **De Leon et al. [36]**	No		NR	NR	NR	>50% estimated vaccine effectiveness by modelling
Israel **Levine et al. [37]**	No	1755 COVID-19 cases after vaccination	NR	NR	NR	Four-fold reduction in SARS-CoV-2 viral load for people developing infections 12–28 days after first dose
Israel **Petter et al. [38]**	No	NR	NR	NR	NR	1.6 to 20x reduction in overall SARS-CoV-2 viral load by vaccinating the community
Israel **Rossman et al. [39]**	No	NR	NR	NR	Pf/B	49% drop in cases, 36% drop in hospitalisations: 1.5 months after vaccine initiation
Scotland **Vasileious et al. [54]**	Yes	1,137,775	34.8% 18–64 65.2% ≥65	61	Pf/B	Estimated efficacy of single dose 85% (Pfizer) and 94% (AstraZeneca) at 28–34 days post vaccination 81% reduction in hospitalisation in those over 80
UK **Hall et al. [55]**	Yes	20,641	16.1% <25 76% 25–64 7.9% ≥65	85	Pf/B (94) AZ/Ox (6)	Vaccine effectiveness at 21 days: 72% after 1 dose, 86% after 2 doses 67% receiving vaccine had previous COVID-19
USA **Bradley, Grundberg, Selvarangan [48]**	No	188 (HCW)	NR	NR	NR	HCW with previous documented SARS-COV-2 infection have higher IgG titres after COVID-19 vaccination

AZ/Ox AstraZenca/Oxford, BB Bharat Biotech, HCW Health care worker, LTCF Long-term care facility, Mod Moderna, NR not reported, Pf/B Pfizer BioNTech, SARS-CoV-2 severe acute respiratory syndrome-coronavirus-2, UK United Kingdom, USA United States of America. * Where data was available for percentage vaccinated by each vaccine type for the study group included.

## 4. Discussion

This rapid systematic review evaluated publicly-available policies and reports for mass-vaccination against COVID-19. Included national policy documents in this study provide an insight into the prioritisation and infrastructure requirements of a COVID-19 mass-vaccination system. These early published findings provide promising evidence of decreases in COVID-19 cases, hospitalisations, and death following vaccine scale-up.

The synthesis of the early national policy documents which are available in the English language provides a framework for the development of future policy documents. One of the most important aspects of vaccine delivery is the equitable access of vaccines to the entire population and vaccinating vulnerable populations to reduce morbidity and mortality [56]. Although Israel has been successful in delivering vaccines to the vast majority of its population, the country is wealthy, well-resourced, and has a small population [9]. The ability to extend this to the Palestinian population remains a challenge [9]. Similarly, vaccinating in settings with larger or vulnerable populations will be challenging. Furthermore, the possibility of shortages to vaccine supply remain an ongoing reality [56]. Although priority groups are clearly stipulated in the policy documents, only two policy documents acknowledge that vaccine shortages may compromise scale-up [21,24].

The national policy documents included in this study illustrate the way in which mass-vaccination campaigns can be implemented in a range of settings. Most countries have been able to rapidly convert large public spaces into vaccination hubs. Other suggested venues for vaccination include hospitals, clinics, places of worship, and nursing homes. Advantages of large venues include the availability of parking spaces and the ability to accommodate large populations [22]. Another option discussed, includes a drive-through parking facility which is used by Qatar, for the second dose, with the first dose administered at a clinic to better facilitate adverse event monitoring [16]. Policies from the UK and New Zealand also provide a framework for recruitment of the increased staffing needs for vaccine delivery via an online volunteer registry [22,26]. The UK policy also details various roles volunteers can have including the military volunteers for logistics and non-trained civilian volunteers as guides and assistance, such as in the car-park [22].

Another challenge with COVID-19 vaccination will be the cost of procurement and delivery of the vaccination process. Only two national policy documents estimate a cost associated with vaccinating the population. The estimated total cost of the vaccination drive in New Zealand is $66 million (US$48million) [26], while in Lebanon the cost of deploying vaccines is budgeted at US$16 million [24]. The estimated cost in Lebanon includes an estimate of supply, training, cold-chain, and other infrastructure requirements for vaccination. It does not include the cost of the vaccines itself; a large portion of which Lebanon plans to procure its vaccines from WHO through the COVAX facility. This is an indication of the expenses associated with an effective vaccination policy. Furthermore, Lebanon has a very small population of 6.8 million, and larger countries will have much greater costs. While Israel has provided a model for vaccination success with the USA, UK, and EU all progressing slowly towards achieving successes with their vaccination drive, middle- and low-income countries will remain vulnerable due to lack of vaccine supply and high infrastructure costs. Another enduring challenge will be the vaccination of refugees and minority groups with uncertain legal status. Although the Lebanese policy, a country where one-third of the population are refugees, discusses the vaccination of refugees [24], in settings where such groups are marginalised or ostracised, reaching out to this population will remain a challenge. Only three national policy documents have stipulated a dedication to providing excess vaccines through COVAX [11,20,22].

The included research articles in this systematic review, confirms the overall safety of vaccines available. Anaphylaxis is rare, indicated by an incidence of <0.001% in large, vaccinated populations in the United States [49,53]. However, when reviewing electronic records of reports of adverse events by individuals presenting to clinicians post-vaccination, the frequency of adverse events was found to be much lower compared to systems that use active surveillance, such as in clinical trials [45]. Furthermore, in the USA, a national passive surveillance system for adverse events in over 13 million vaccinated individuals found the incidence of adverse events to be a 0.05% [44]. The true incidence of serious adverse-effects however need to be determined by studies that are appropriately statistically powered and through large meta-analysis. This can assist in planning scale-up of vaccination, to reassure the populations of the safety of vaccinations and to plan extra health care staff required for adverse event management.

There were five studies which evaluated vaccine acceptance [46,47,50,51,52]. All studies involved HCW. Factors associated with low vaccine uptake include younger age [50], female sex [51] and black populations [47,52]. Higher rates of acceptance was associated with higher income and education levels in one setting [52]. Effective health marketing, which is tailored to and reaches younger people, females, as well as ethnically/linguistically diverse populations should be incorporated into national vaccination strategies.

This study has several limitations. The restriction to English language sources resulted in a high proportion of included reports and policies being from high-income countries. Only one policy was identified from an upper middle-income country. Furthermore, vaccination outcomes in countries with large, COVID-19 affected populations such as China, India, Russia, and Brazil were not included due to the language restriction. Vaccines against COVID-19 only became commercially available three months prior to this review; hence, this study reports only the early experiences with mass-vaccination. Further updated reviews should be undertaken as scale-up proceeds.

Based on the findings in this study we recommend the following strategies for countries up-scaling or commencing the vaccination process:

- Vaccinate health care workers, elderly, and those with chronic comorbidities as a priority.
- Utilise mass vaccination hubs such as sporting venues, for maximal scale and efficiency.
- Consider novel delivery techniques such as drive-through clinics for the second dose, where this is feasible and culturally acceptable.
- Consider use of automated electronic surveys for monitoring side-effects among all recipients.
- Engage the community to increase vaccination awareness and acceptance including by
  ○ Including and training volunteers in the vaccination effort.
  ○ Using social media and media campaigns to raise awareness.
  ○ Having a means to monitor mis-information.
  ○ Utilising high profile "champions", such as political leaders and social celebrities. Support COVAX and global commitments to deliver vaccines to marginalised and vulnerable populations, because everyone deserves to be protected and the global population cannot be safe until we are all vaccinated.

Despite these recommendations, it is important to remember that a vaccination strategy alone may not curb the COVID-19 pandemic. Ongoing infection control and social distancing must remain in place until long term solutions are established. Furthermore, although the perceived risk is low, with no evidence of long-term adverse effects in animal models and limited biological plausibility in humans, prior to the current pandemic, no mRNA or DNA based vaccines authorised for use and long-term effects of use of novel vaccination techniques remain unknown. Research into drug therapy remains important including new and repurposed therapy active against SARS-CoV-2 [57,58].

## 5. Conclusions

This study summarises data from early mass-vaccination programmes against COVID-19 and offers insights into those initial efforts as a contribution to important debates about strategy and approach globally. It highlights strategies that can be used to improve scale-up, equitable delivery, and vaccine acceptance in support of ongoing vaccination effort by governments and public health bodies around the world.

## Data Availability

No new data were created or analyzed in this study. Data sharing is not applicable to this article.

## Supplementary Materials

The following are available online at https://www.mdpi.com/article/10.3390/vaccines9040326/s1, Table S1: Overview of COVID-19 vaccination data available on government websites in English.
