# Supplementary material for: The Implementation of Mass-Vaccination against SARS-CoV-2: A Systematic Review of Existing Strategies and Guidelines
Source: Vaccines (Basel). 2021 Apr 1;9(4):326. doi: 10.3390/vaccines9040326 (PMC8066252; doi:10.3390/vaccines9040326)
Supplement: Supplementary file 1 [file vaccines-09-00326-s001.pdf]

Table S1. Overview of COVID-19 vaccination data available on government websites in English.

Country	Austria	Belgium	Canada	EU	Iceland	India	Ireland	Lebanon	Nepal	Netherlands	Seychelles	Switzerland	UAE	UK	USA
Reference	[1]	[2]	[3]	[4]	[5]	[6]	[7]	[8]	[9]	[10]	[11]	[12]	[13]	[14]	[15]
Date of document	Feb 28	Feb 28	Feb 26	Feb 24	Feb 26	Feb 23	Feb 25		Feb 21	Feb 23	Feb 26	Feb 26	Feb 28	Feb 28	Feb 28
Date of first vaccine in setting	10 January 2021		19 December 2020		19 December 2020	16 January 2021		15 February 2021		6 January 2021				13 December 2020	
Total population of setting (million)	9.0	11.6	37.7	444.9	0.3	1380	4.9	6.8	29.1	17.1	0.1	8.7	9.9	67.9	331
Vaccines offered	Pf/B	Pf/B Mod AZ/Ox	Pf/B Mod	NR	Pf/B Mod AZ/Ox	NR	Pf/B Mod AZ/Ox	Pf/B	NR	Pf/B Mod AZ/Ox	Az/Ox Sinopharm	NR	NR	NR	Pf/B Mod
Total number of vaccine doses administered ('000)	648,639	1,106,512	1,777,405	27,425,844	32,157	11,900,000	NR	40,641	NR	1,319,008	75,096	748,791	6,020,461	NR	75,200,000
Total number of individuals vaccinated ('000)	414,441	800,030	1,086,025	NR	NR	NR	NR	NR	NR	NR	NR	NR	NR	NR	NR
Percentage vaccinated—total (%) *	5.3	7.0	2.9	NR	NR	NR	8.3	NR	1.4	7.0	52	NR	NR	29.6	15
Percentage of the population receiving one dose (%) *	2.7	4.3	1.7	NR	NR	NR	5.5	NR	NR	4.7	28	NR	NR	28.5	7.5
Percentage of population receiving two doses (%) *	2.6	2.7	1.1	NR	NR	NR	2.8	NR	NR	1.3	24	2.5	NR	1.1	7.5
Percentage of all eligible males vaccinated (%)	2.3	NR	4.0	NR	NR	NR	NR	NR	NR	NR	NR	NR	NR	NR	NR
Percentage of all eligible females vaccinated (%)	6.1	NR	1.7	NR	NR	NR	NR	NR	NR	NR	NR	NR	NR	NR	NR

Percentage of those in each age group vaccinated (%)		NR		NR	NR	NR	NR	NR	NR	NR	NR	NR	NR	NR	NR
Under 18 years	0.07		0.03												
18–65 years	9.28		1.6–4.7												
>65 years	29.55														
Percentage of those in high risk groups vaccinated (%)	NR	NR		NR	NR	NR	NR	NR	NR	NR	NR	NR	NR	NR	NR
Frontline HCW			47.8												
Aged care residents			67.5												
Elderly			14.1												
Time taken to vaccinate first 100,000 people (days)	NR	NR	NR	NR	NR	NR	NR	NR	NR	34	NR	NR	NR	14	NR
Time taken to vaccinate first 1,000,000 people (days)	NR	18	24	NR	NR	NR	NR	NR	NR	NR	NR	NR	NR	NR	NR

AZ/Ox AstraZenca/Oxford, EU European Union, HCW Health care worker, Mod Moderna, NR not reported, Pf/B Pfizer BioNTech, UAE United Arab Emirates, UK United Kingdom, USA United States of America. * may be calculated value.

Reference

1. Federal Ministry Republic of Austria. *COVID-19 Vaccination in Austria*; Federal Ministry Republic of Austria: Vienna, Austria, 2021. Available online: https://info.gesundheitsministerium.at/en/ (accessed on 1 March, 2021).
2. datrix.be. Covid Vaccinations Belgium. 2021. Available online: https://covid-vaccinatie.be/en (accessed on 1 March, 2021).
3. Government of Canada. COVID-19 Vaccination in CANADA. 2021. Available online: https://health-infobase.canada.ca/covid-19/vaccine-administration/ (accessed on 1 March, 2021).
4. European Centre for Disease Prevention and Control. *Overview of the Implementation of COVID-19 Vaccination Strategies and Vaccine Deployment Plans in the EU/EEA*; European Centre for Disease Prevention and Control: Stockholm, Sweden, 2021.
5. The Directorate of Health and the Department of Civil Protection and Emergency Management. *COVID-19 Vaccinations in Iceland—Statistics*; The Directorate of Health and the Department of Civil Protection and Emergency Management: *Reykjavík*, Iceland, 2021. Available online: https://www.covid.is/statistical-information-on-vaccination (accessed on 1 March, 2021).
6. Government of India. Update on COVID-19 Vaccination-Day 39. 2021. Available online: https://pib.gov.in/PressReleseDetailm.aspx?PRID=1700269 (accessed on 1 March, 2021).
7. Government of Ireland. Ireland's COVID-19 Data. 2021. Available online: https://covid19ireland-geohive.hub.arcgis.com (accessed on 1 March, 2021).
8. Central Inspection Lebanon. 2021 Available online: https://impact.cib.gov.lb/home/dashboard/vaccine (accessed on 1 March, 2021).

9. Health Sector Response to COVID-19. Government of Nepal. Health Sector Response to COVID-19, Nepal. 2021. Available online: https://covid19.mohp.gov.np/ (accessed on 1 March, 2021).
10. National Institute for Public Health and the Environment. *Figures on the COVID-19 Vaccination Programme*; National Institute for Public Health and the Environment: Utrecht, The Netherlands, 2021. Available online: https://www.rivm.nl/en/covid-19-vaccination/figures-on-covid-19-vaccination-programme (accessed on 1 March, 2021).
11. Ministry of Health Seychelles. *COVID-19 Vaccination Uptake Report*; Ministry of Health Seychelles: Victoria, Seychelles, 2021.
12. Federal Office of Public Health FOPH. COVID-19 Switzerland. Federal Office of Public Health FOPH. 2021. Available online: https://www.covid19.admin.ch/en/epidemiologic/vacc-doses?detGeo=CH&vaccRel=abs&geoView=table (accessed on 1 March, 2021).
13. Ministry of Health and Prevention. *UAE Coronavirus (COVID-19) Updates*; Ministry of Health and Prevention, United Arab Emirates, 2021. Available online: covid19.ncema.gov.ae/en (accessed on 1 March, 2021).
14. Gov.UK. *Coronavirus (COVID-19) in the UK*; Gov.UK, United Kingdom, 2021. Available online: https://coronavirus.data.gov.uk/details/vaccinations (accessed on 1 March, 2021).
15. Center for Disease Control and Prevention. *COVID-19 Vaccinations in the United States*; Center for Disease Control and Prevention: Atlanta, GA, USA, 2021. Available online: https://covid.cdc.gov/covid-data-tracker/#vaccinations (accessed on 1 March, 2021).
